# Supplementary material for: Structure of native chromatin fibres revealed by Cryo-ET in situ
Source: Nat Commun. 2023 Oct 10;14:6324. doi: 10.1038/s41467-023-42072-1 (PMC10564948; doi:10.1038/s41467-023-42072-1)
Supplement: Supplementary file 1 — Supplementary Information [file 41467_2023_42072_MOESM1_ESM.pdf]

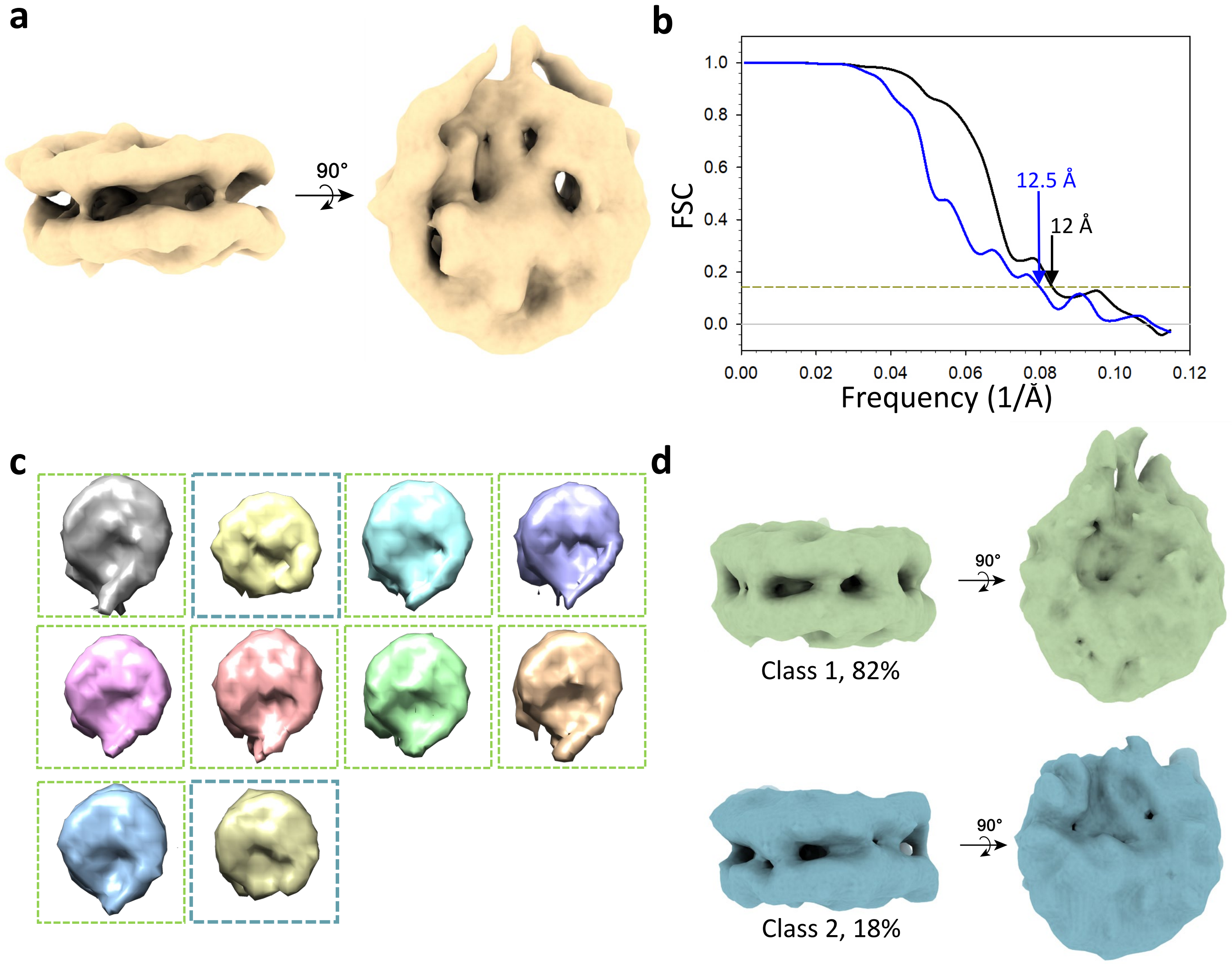

Supplementary Figure 1 | Subtomogram averaging and classification of native nucleosomes. a) A subtomogram average of native nucleosomes *in situ* (from n = 6,790, n of tomograms = 5). Two orthogonal views are shown. **b)** Gold-standard Fourier shell correlation (FSC) curves of subtomogram averaged maps from all nucleosomes (black line) and from Class 1 (blue line). **c)** Classification of 6,790 native nucleosome particles. Nucleosome particles from classes framed by dashed green lines showing prominent linker DNA density and partial H1 density are combined into Class 1, whereas nucleosome particles from classes framed by light blue lines corresponding to the canonical core nucleosome structure are combined into Class 2. **d)** Subtomogram averages of Class 1 nucleosomes (from n = 5,578) (top) and Class 2 native nucleosome (from n = 1,212) (bottom), shown in two views.
