# Supplementary material for: Structure of native chromatin fibres revealed by Cryo-ET in situ
Source: Nat Commun. 2023 Oct 10;14:6324. doi: 10.1038/s41467-023-42072-1 (PMC10564948; doi:10.1038/s41467-023-42072-1)
Supplement: Supplementary file 3 — Description of Additional Supplementary Files [file 41467_2023_42072_MOESM3_ESM.pdf]

File name: Supplementary Movie 1

Description: A representative reconstructed tomogram with the segmentation of nuclear envelope, ribosomes, and nucleosomes. The tomogram is reconstructed with SIRT-like filtering in IMOD 4.11 and sliced along the Z axis back and forth through slices 1 to 269. The segmented volume is overlapped with the tomogram in ChimeraX, NE, ribosomes and nucleosomes are coloured grey, blue and gold, respectively. An enlarged view of the nucleosome is introduced at the end.

File name: Supplementary Movie 2

Description: *In-situ* structure of the native nucleosome fitted with the crystal model. The transparency of the *in-situ* structure is adjusted to 50% and the crystal model (PDB 6ESF) is colored grey. The spinning is 360° and conducted in ChimeraX.

File name: Supplementary Movie 3

Description: Representative tomographic slices of native chromatin fibres. The reconstructed tomogram is fixed at -16° of the Y axis and rotated back and forth along the X axis from -15° to +15° in IMOD 4.11.
