# Supplementary material for: Structure of native chromatin fibres revealed by Cryo-ET in situ
Source: Nat Commun. 2023 Oct 10;14:6324. doi: 10.1038/s41467-023-42072-1 (PMC10564948; doi:10.1038/s41467-023-42072-1)
Supplement: Supplementary file 7 — Reporting Summary [file 41467_2023_42072_MOESM7_ESM.pdf]

nature portfolio

Corresponding author(s): Peijun Zhang

Last updated by author(s): Sep 20, 2023

Reporting Summary

Nature Portfolio wishes to improve the reproducibility of the work that we publish. This form provides structure for consistency and transparency in reporting. For further information on Nature Portfolio policies, see our Editorial Policies and the Editorial Policy Checklist.

Statistics

For all statistical analyses, confirm that the following items are present in the figure legend, table legend, main text, or Methods section.

n/a	Confirmed	
☐	☒	The exact sample size (*n*) for each experimental group/condition, given as a discrete number and unit of measurement
☐	☒	A statement on whether measurements were taken from distinct samples or whether the same sample was measured repeatedly
☒	☐	The statistical test(s) used AND whether they are one- or two-sided<br>*Only common tests should be described solely by name; describe more complex techniques in the Methods section.*
☒	☐	A description of all covariates tested
☒	☐	A description of any assumptions or corrections, such as tests of normality and adjustment for multiple comparisons
☒	☐	A full description of the statistical parameters including central tendency (e.g. means) or other basic estimates (e.g. regression coefficient) AND variation (e.g. standard deviation) or associated estimates of uncertainty (e.g. confidence intervals)
☒	☐	For null hypothesis testing, the test statistic (e.g. *F*, *t*, *r*) with confidence intervals, effect sizes, degrees of freedom and *P* value noted<br>*Give P values as exact values whenever suitable.*
☒	☐	For Bayesian analysis, information on the choice of priors and Markov chain Monte Carlo settings
☒	☐	For hierarchical and complex designs, identification of the appropriate level for tests and full reporting of outcomes
☒	☐	Estimates of effect sizes (e.g. Cohen's *d*, Pearson's *r*), indicating how they were calculated

Our web collection on statistics for biologists contains articles on many of the points above.

Software and code

Policy information about availability of computer code

Data collection	Cryo-ET data were collected using Tomography Software V5.0
Data analysis	Cryo-ET data processing: IMOD(v4.11), emClarity (v1.5.0.2 and v1.5.3.10), RELION (v4.0), MagpiEM [https://github.com/fnight128/MagpiEM].

For manuscripts utilizing custom algorithms or software that are central to the research but not yet described in published literature, software must be made available to editors and reviewers. We strongly encourage code deposition in a community repository (e.g. GitHub). See the Nature Portfolio guidelines for submitting code & software for further information.

Data

Policy information about availability of data

All manuscripts must include a data availability statement. This statement should provide the following information, where applicable:

- Accession codes, unique identifiers, or web links for publicly available datasets
- A description of any restrictions on data availability
- For clinical datasets or third party data, please ensure that the statement adheres to our policy

The cryoET subtomogram averaging maps of in situ nucleosome structures in this study are available in the EMDB database under the accession codes EMD-16978 [https://www.ebi.ac.uk/emdb/EMD-16978] (in situ H1-bound nucleosome structure with linker DNA), EMD-16979 [https://www.ebi.ac.uk/emdb/EMD-16979] (in situ H1-bound nucleosome structure with linker DNA) and EMD-16980 [https://www.ebi.ac.uk/emdb/EMD-16980] (in situ nucleosome structure without linker DNA).

Research involving human participants, their data, or biological material

Policy information about studies with human participants or human data. See also policy information about sex, gender (identity/presentation), and sexual orientation and race, ethnicity and racism.

Reporting on sex and gender	N/A
Reporting on race, ethnicity, or other socially relevant groupings	N/A
Population characteristics	N/A
Recruitment	N/A
Ethics oversight	N/A

Note that full information on the approval of the study protocol must also be provided in the manuscript.

Field-specific reporting

Please select the one below that is the best fit for your research. If you are not sure, read the appropriate sections before making your selection.

☒ Life sciences ☐ Behavioural & social sciences ☐ Ecological, evolutionary & environmental sciences

For a reference copy of the document with all sections, see nature.com/documents/nr-reporting-summary-flat.pdf

Life sciences study design

All studies must disclose on these points even when the disclosure is negative.

Sample size	For cryo-ET subtomogram averaging, 5 tomograms from 5 lamellae were selected. The 5 tomorgams were chosen based on the thickness, which is critical for the determination of in situ strutures as thin tomograms yield high signal to noise ratio.
Data exclusions	No data were excluded in this study.
Replication	For cryo-ET subtomogram averaging, subtomograms were extracted from 5 discrete tomograms of 5 different cells based on the template matching results. Two randomly divided half datasets were independently processed and combined to give rise to the final cryoET subtomogram averaging map. The final resolution was assessed by comparing the two independent maps. These results can be reproduced and there are no findings that cannot be reproduced.
Randomization	The subtomograms were randomly divided into ODD and EVEN datasets, as standard approach implemented in emClarity (v1.5.0.2 and v1.5.3.10) and RELION (v4.0).
Blinding	Quantification of chromatin fibres and nucleosomes were not blinded as the fibres could be clearly identified and the nuclear envelope was identified to exclude false positives of nucleosomes for subtomogram averaging.

Reporting for specific materials, systems and methods

We require information from authors about some types of materials, experimental systems and methods used in many studies. Here, indicate whether each material, system or method listed is relevant to your study. If you are not sure if a list item applies to your research, read the appropriate section before selecting a response.

Materials & experimental systems

n/a	Involved in the study	
☒	☐	Antibodies
☐	☒	Eukaryotic cell lines
☒	☐	Palaeontology and archaeology
☒	☐	Animals and other organisms
☒	☐	Clinical data
☒	☐	Dual use research of concern
☒	☐	Plants

Methods

n/a	Involved in the study	
☒	☐	ChIP-seq
☒	☐	Flow cytometry
☒	☐	MRI-based neuroimaging

April 2023

Eukaryotic cell lines

Policy information about cell lines and Sex and Gender in Research

Cell line source(s)	CEM CD4+ T-cells from human T lymphoblastic leukemia (catalogue ARP-117, HIV reagents Program). Primary cell lines were derived from a female human participant.
Authentication	CEM CD+ T-cells are authenticated by ATCC.
Mycoplasma contamination	All the cells were tested negative for mycoplasma contamination.
Commonly misidentified lines (See ICLAC register)	N/A
